# Association analysis of color characteristics, anthocyanin profiles, and antioxidant capacity in purple/black agricultural products based on targeted metabolomics

**DOI:** 10.1016/j.fochx.2026.104268

**Published:** 2026-08-02

**Authors:** Xiao Ren, Jianfeng Pu, Ying Nie, Yuchen Wang, Shuxian Huang, Dazhou Zhu

**Affiliations:** a Institute of Food and Nutrition Development, Ministry of Agriculture and Rural Affairs, Beijing 100081, China; b Rural Revitalization College, Zhaotong Vocational College, Zhaotong 657000, China

**Keywords:** Electronic eye analysis, Pigment composition, UPLC–MS/MS, In vitro antioxidant capacity, Chromatic–pigment–antioxidant relationship

## Abstract

This study systematically compared color traits, pigment composition, anthocyanin profiles, and antioxidant capacity in 16 representative purple/black agricultural products using electronic eye analysis, HPLC, and targeted UPLC–MS/MS. Pigment distribution, content, and composition differed markedly among samples. Despite similar surface appearance, homogenized samples varied in color, with 10 appearing purple, 4 yellowish, and 2 greenish. Anthocyanins predominated in the 14 purple and yellowish homogenized samples, with the highest total contents in blueberry (1441.13 μg/g) and blackberry (1029.62 μg/g). By contrast, chlorophyll A predominated in black soybean and purple lablab bean, the two greenish homogenates. A total of 92 anthocyanins were identified, with purple grape showing the greatest diversity. Cyanidin-type derivatives, especially cyanidin-3-O-glucoside, were the most widespread. Total anthocyanins were negatively associated with L* and C* values but positively associated with antioxidant capacity, indicating greater antioxidant potential in darker samples. These findings support color-based dietary guidance.

## Highlights

• Color, pigments and antioxidants were compared across 16 purple/black samples.
• Pigment localization made homogenized color traits differ from surface appearance.
• UPLC–MS/MS quantified anthocyanin fingerprints in all samples.
• Cyanidin-type anthocyanins were most widespread, especially cyanidin-3-O-glucoside.
• Total anthocyanins negatively related to L* and C* but positively to antioxidants.

## Graphical abstract

**Figure:**
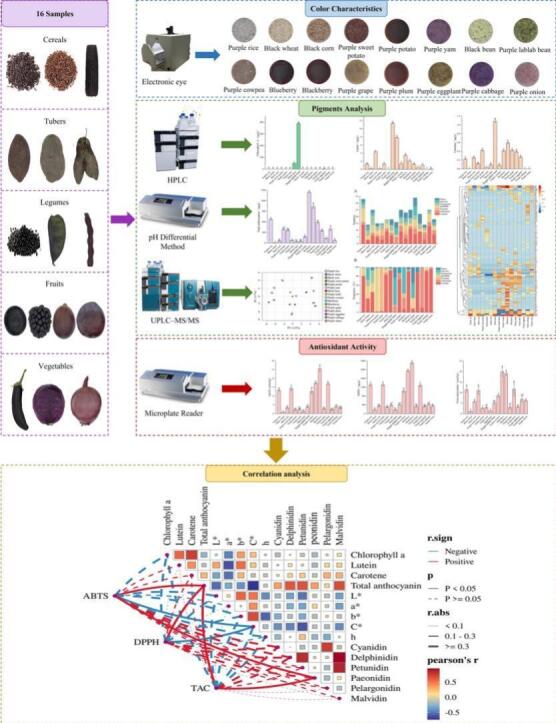

## 1 Introduction

With the increasing diversification of food supply, consumers are confronted with a more complex food environment, which may make it more difficult to make dietary choices that align with their individual nutritional needs (Vermeulen, Park, Khoury, & Bene, 2020). Color is a critical attribute of agricultural products, which significantly affects consumer preferences and purchasing decisions (Pathare, Opara, & Al-Said, 2012). The pigments present in agricultural products not only impart characteristic coloration and enhance sensory appeal, but also serve physiological functions and possess considerable nutritional value (Kumar, Kumar, & Shekhar, 2020). In this context, the field of nutritional science has proposed the concept of “color nutrition,” which encourages the intake of foods of various colors to ensure comprehensive nutrient acquisition (De Mejia, Zhang, Penta, Eroglu, & Lila, 2020). Some studies classify foods into five color groups (red, orange, yellow, green, and purple/black), which purple/black foods are widely represented across categories such as cereals, tubers, legumes, fruits, and vegetables. These foods are rich in functional compounds such as polyphenols, which are associated with improved cognition, memory, and emotional well-being (Minich, 2019). Purple/black agricultural products have attracted significant interest due to their rich variety and high nutritional value (Francavilla & Joye, 2020; Nuszkiewicz, Wroblewska, Wroblewski, & Wozniak, 2025). However, the quantitative relationships among their coloration, pigment composition, and nutritional content remain inadequately understood.

Foods such as blueberries, black rice, and purple sweet potatoes typically exhibit a purple/black appearance mainly due to their high contents of anthocyanins, which are a major class of natural pigments responsible for colors ranging from orange-red to blue-purple (Giusti & Wrolstad, 2003). In some purple/black agricultural products, other pigments, such as carotenoids and chlorophylls, may also contribute to the final coloration (Jo et al., 2021; Kaur & Kapoor, 2002; Tanaka, Sasaki, & Ohmiya, 2008). Anthocyanidins are commonly classified into six principal types: cyanidin, delphinidin, petunidin, peonidin, pelargonidin, and malvidin (Pojer, Mattivi, Johnson, & Stockley, 2013). These anthocyanidins serve as aglycone cores of anthocyanins, which are further modified by glycosylation and, in some cases, acylation. Structural modifications arising from hydroxylation, methylation, glycosylation, and acylation generate hundreds of anthocyanin derivatives with diverse physicochemical properties and biological activities (Bueno et al., 2012). The core chromophore of anthocyanins is the 7-hydroxyflavylium cation. It comprises an extended π-conjugated system with eight conjugated double bonds and a positively charged oxygen-containing heterocycle. When the B-ring is enriched in methoxy substituents, this structure typically produces an intense red–orange coloration. Conversely, an increasing degree of hydroxylation on the B-ring intensifies the blue–purple hue of anthocyanin pigments (Khoo, Azlan, Tang, & Lim, 2017). Structural differences among anthocyanidins—specifically the number and position of hydroxyl and methoxy groups—determine variations in their light absorption spectra, thereby contributing to a wide color range from orange–red to violet–blue (Glover & Martin, 2012). For example, pelargonidin-derived anthocyanins generally confer orange–red hues, cyanidin derivatives are associated with red–purple coloration, whereas delphinidin-based anthocyanins typically exhibit deep purplish–blue tones (Glover & Martin, 2012). Beyond the aglycone structure, variation in glycosylation (e.g., conjugation with glucose, galactose, rhamnose, or rutinose) can further shift the absorption maximum, resulting in bathochromic (blue) or hypsochromic (red) shifts (Shibata, Shibata, & Kasiwagi, 1919). Current research on the coloration of purple/black agricultural products has primarily focused on the identification and quantification of anthocyanin species within individual species (Abdel-Aal, Young, & Rabalski, 2006; Mock, Hartmann, Dehmer, & Martens, 2017; Veberic, Slatnar, Bizjak, Stampar, & Mikulic-Petkovsek, 2015; Zhu et al., 2022). However, substantial differences in anthocyanin extraction methods, analytical instrumentation, and experimental conditions among studies, hinder direct comparisons of anthocyanin composition and contents across species.

Beyond their contribution to coloration, anthocyanins exhibit strong in vitro antioxidant activity. Purple, blue, and red fruits and vegetables rich in anthocyanins are categorized as high-antioxidant foods and may contribute over 20% of the recommended daily antioxidant intake (Comert, Mogol, & Gokmen, 2020). Anthocyanins can efficiently scavenge free radicals and interrupt chain reactions associated with oxidative damage (He & Giusti, 2010), thereby playing important roles in health promotion, including vision protection, obesity prevention, and the mitigation of cardiovascular diseases and cancer (Chen, Xu, Sun, Jiang, & Bai, 2022; Garcia & Blesso, 2021; Liu et al., 2012; Ma, Du, Li, Yang, & Zhu, 2021). Their antioxidant activity is determined not only by total concentration but also by structural features, including anthocyanidin type, glycosylation sites, modification patterns, and overall molecular composition (Koss-Mikolajczyk & Bartoszek, 2023). For example, anthocyanins with a greater number of hydroxyl groups on the B-ring exhibit stronger antioxidant properties (Dudek, Spiegel, Strugala-Danak, & Gabrielska, 2022). Simpler anthocyanin structures exhibit higher antioxidant potency, monosaccharide-conjugated anthocyanins show stronger antioxidant activity than those conjugated with polysaccharides, and glycosylated forms tend to be more active than acylated ones (Gao, Li, Li, Liang, & Zhang, 2022). Current research primarily focuses on the nutritional functionality of individual agricultural products (Gamel, Saeed, Ali, & Abdel-Aal, 2023; Stull et al., 2024; Xu et al., 2024). However, further investigation is needed to clarify differences in antioxidant activity among various types of purple/black agricultural products, as well as to elucidate the relationships among color appearance, anthocyanin composition, and antioxidant capacity.

In this study, a total of 16 representative purple/black agricultural products were selected, including purple rice, black wheat, black corn, purple sweet potato, purple potato, purple yam, black soybean, purple lablab bean, purple cowpea, blueberry, blackberry, purple grape, purple plum, purple eggplant, purple cabbage, and purple onion. Their color characteristics were systematically analyzed using electronic eye (*E*-eye) technology. Furthermore, high-performance liquid chromatography (HPLC) and liquid chromatography–mass spectrometry (LC-MS) were employed to qualitatively and quantitatively analyze the composition and concentration of the major color-contributing compounds in all samples. Antioxidant capacity was also evaluated, and comprehensive correlation analyses were conducted to explore the relationships among color parameters, the composition and content of chromogenic compounds, and antioxidant activity. Compared with previous studies on individual samples, this study provides a systematic cross-category evaluation of 16 representative purple/black agricultural products using a unified analytical framework, thereby offering new insight into the relationships among color traits, pigment composition, and antioxidant potential.

## 2 Materials and methods

### 2.1 Samples and chemicals

All agricultural products used in this study and their sources are listed in Table S1. All samples were processed within 3 d after purchase, and a portion of each sample was ground in liquid nitrogen and stored at −80 °C until future analysis. Standards of chlorophyll A (purity ≥95%), chlorophyll B (purity ≥95%) and β-carotene (purity ≥98%), as well as 2,2′-Azino-bis (3-ethylbenzothiazoline-6-sulfonic acid) (ABTS) and 1,1-Diphenyl-2-picry lhydrazyl radical (DPPH) assay kits, were purchased from Solarbio Science & Technology Co., Ltd. (Beijing, China). The total antioxidant capacity (TAC) assay kit (ferric reducing antioxidant power (FRAP) method) was obtained from Shanghai Yuanye Bio-Technology Co., Ltd. (Shanghai, China). Oasis HLB solid-phase extraction cartridges (60 mg) were obtained from Waters (Barcelona, Spain). Ascorbic acid, protease, α-amylase, acetonitrile and methanol were purchased from Sigma–Aldrich (Shanghai, China). All chemical reagents used in this study were of analytical grade.

### 2.2 Color characteristics analysis

Color parameters of the samples were determined using an electronic eye system (DigiEye, VeriVide, UK). Samples were photographed and the CIE L* (lightness), a* (red/green), b* (yellow/blue), C* (chroma) and h* (hue angle) values were recorded. For each sample type, three independent biological replicates were analyzed. For each replicate, images of the whole homogenized sample, the intact surface, and the transverse section were acquired, and five measurements were taken. All measurements were carried out at room temperature (25 ± 1 °C).

### 2.3 Determination of chlorophyll content

Chlorophyll A and chlorophyll B were quantified according to the method of Zhang et al. (Zhang et al., 2025) with slight modifications. Briefly, frozen powdered samples (1.0 g) were accurately weighed and extracted with 15 mL of 0.1% (*w*/*v*) 2,6-di-tert-butyl-4-methylphenol (BHT) in methanol using an ultrasonic bath for 2 min. The mixture was then centrifuged at 8000 r/min for 5 min and the supernatant was collected. The residue was re-extracted twice under the same conditions, and the supernatants were combined. The combined extract was evaporated to dryness under a gentle stream of nitrogen and reconstituted in 5 mL of 0.1% (w/v) BHT in methanol. The solution was passed through a 0.45 μm organic membrane filter prior to high-performance liquid chromatography (HPLC) analysis using a Shimadzu HPLC system (Kyoto, Japan) equipped with a UV detector (SPD-20 A, Shimadzu, Japan) and a ZORBAX SB-C18 column (150 × 4.6 mm, Agilent, USA). Isocratic elution was carried out with methanol as the mobile phase at a flow rate of 1.0 mL/min. Chlorophyll A and chlorophyll B were detected at 665 nm and 449 nm.

### 2.4 Determination of carotenoid content

β-Carotene was extracted and quantified according to the National Standard of the People's Republic of China GB 5009.83-2016, with minor modifications. Briefly, a 2.0 g portion of frozen powdered samples (purple rice, black wheat, black maize, purple sweet potato, purple potato, and black soybean) were weighed and mixed with 1.0 g of ascorbic acid, 15 mL of deionized water, 0.5 g of protease, and 0.5 g of α-amylase to account for the high protein or starch content. The mixture was incubated at 55 °C with shaking for 30 min. For samples with relatively low protein or starch content, a 2.0 g portion of frozen powder was directly mixed with 1.0 g of ascorbic acid without enzyme treatment.

Subsequently, absolute ethanol (75 mL) was added to all samples, and the mixtures were incubated at 60 °C for 30 min. Then, potassium hydroxide solution (50%, *w*/w; 25 mL) was added, and saponification was performed at 53 °C for 30 min. After cooling to room temperature, the mixtures were extracted twice with petroleum ether. The combined organic layers were dried over anhydrous sodium sulfate and evaporated to dryness under a nitrogen stream. The resulting residues were re-dissolved in 5 mL of dichloromethane and filtered through a 0.45 μm membrane filter before HPLC analysis.

β-Carotene quantification was carried out using an HPLC system (U3000, Dionex, Sunnyvale, CA, USA) equipped with a C18 reversed-phase column (250 × 4.6 mm, 5 μm; Agilent Technologies, USA) and a UV detector. The mobile phase consisted of chloroform, acetonitrile, and methanol in a volume ratio of 3:12:85, supplemented with 0.4 g/L ascorbic acid. The flow rate was maintained at 1.0 mL/min, and β-carotene was detected at 450 nm.

### 2.5 Anthocyanins analysis

#### 2.5.1 Extraction of anthocyanins

Anthocyanins were extracted according to a previously reported method (Fan, Lin, Zhang, Li, & Tang, 2023) with slight modifications. Briefly, a 2.0 g portion of frozen powdered samples was mixed with 8 mL of acidified methanol (1%, v/v) and extracted by ultrasonication (200 W, 24 kHz) at 40 °C for 10 min. The mixture was then centrifuged at 10,000 rpm for 7 min, and the supernatant was collected. The residue was re-extracted twice under the same conditions. All supernatants were combined, evaporated to dryness under a gentle stream of nitrogen, and reconstituted with 1% (v/v) acidified aqueous solution to a final volume of 10 mL.

The anthocyanin solution was further purified using Oasis HLB SPE cartridges. The cartridges were preconditioned sequentially with 6 mL methanol and 6 mL water. Then, the sample solution (10 mL) was loaded onto the cartridge, followed by washing with 12 mL water to remove interfering substances. Anthocyanins were eluted under gravity with 9 mL of 1% (v/v) acidified methanol, and the eluate was adjusted to 10 mL. The purified anthocyanin extracts were stored at −80 °C until further analysis.

#### 2.5.2 Determination of total anthocyanin content

Total anthocyanin content was determined using the pH differential method described by Geng et al. (2024), with minor modifications. Briefly, the samples (40 μL) were mixed with the potassium chloride (pH = 1.0) and sodium acetate (pH = 4.5) buffers (160 μL) in a 96-well plate respectively. The mixtures were equilibrated in the dark for 15 min, and the absorbance at 510 and 700 nm was measured using a microplate reader (SpectraMax 340PC, Molecular Devices, USA). total anthocyanin content was expressed as cyanidin-3-O-glucoside equivalents and calculated using the following equation:

$$A={\left(A_{510\mathrm{nm}}-A_{700\mathrm{nm}}\right)}_{\mathrm{pH}\;1.0}-{\left(A_{510\mathrm{nm}}-A_{700\mathrm{nm}}\right)}_{\mathrm{pH}\;4.5}$$

$$\mathrm{Total anthocyanin content}\;\left(\mathrm{\mu g}/g\right)=\frac{A\times \mathrm{MW}\times \mathrm{DF}\times V\times 1000}{ɛ\times l\times m}$$

where A is the absorbance as defined above; MW is the molecular weight of cyanidin-3-O-glucoside (449.2 g/mol); DF is the dilution factor; V is the the total volume of the extract (mL); ε is the molar extinction coefficient of cyanidin-3-O-glucoside (26,900 L/mol·cm); l is the optical path length (typically 1 cm); and m is the mass of the sample (g).

#### 2.5.3 UPLC–MS/MS analysis

Anthocyanin profiling was performed using an ultra-performance liquid chromatography–tandem mass spectrometry (UPLC–MS/MS) system comprising an ExionLC™ AD UPLC (AB Sciex, USA) coupled with a QTRAP® 6500+ triple quadrupole–linear ion trap mass spectrometer equipped with an electrospray ionization (ESI) source. Chromatographic separation was achieved using a Waters ACQUITY BEH C18 column (100 mm × 2.1 mm i.d., 1.7 μm). The mobile phase consisted of solvent A (ultrapure water with 0.5% formic acid, *v*/v) and solvent B (methanol with 0.5% formic acid, v/v). The gradient elution program was as follows: 0.00 min, 5% B; 0.00–6.00 min, linear increase to 50% B; 6.00–12.00 min, linear increase to 95% B, held for 2 min; at 14.00 min, returned to 5% B and equilibrated for 2 min. The flow rate was 0.35 mL/min, the column temperature was maintained at 40 °C, and the injection volume was 2 μL.

Mass spectrometric detection was performed in positive ion mode. The electrospray ionization (ESI) source was operated at 550 °C with an ion spray voltage of 5500 V. The curtain gas (CUR) pressure was maintained at 35 psi. Quantitative analysis of anthocyanins was conducted in multiple reaction monitoring (MRM) mode using compound-specific optimized parameters, including declustering potentials (DP) and collision energies (CE) for each precursor-product ion pair. Data acquisition was performed using Analyst software (version 1.6.3, AB Sciex, USA), and quantitative data processing was carried out using MultiQuant software (version 3.0.3, AB Sciex, USA).

### 2.6 Antioxidant activity

The antioxidant activities of the different agricultural products were assessed using ABTS, DPPH, and total antioxidant capacity (TAC) assay kit (FRAP method) assay kits, following the manufacturers' protocols. All assays were conducted in accordance with the respective instructions provided with the commercial kits.

### 2.7 Statistical analysis

All experiments were performed with at least three independent biological replicates. Data are presented as mean ± standard deviation (SD). One-way analysis of variance (ANOVA) was employed to determine statistically significant differences among groups. Statistical analyses were carried out using SPSS software (version 26.0; SPSS Inc., Chicago, IL, USA), and differences were considered statistically significant at *p* < 0.05. Graphs were generated using Origin software (version 2024; OriginLab Corp., Northampton, MA, USA).

## 3 Results and discussion

### 3.1 Color characteristics of purple/black agricultural products

In the CIE LAB color space, the L* value represents lightness, ranging from 0 (black) to 100 (white); the a* describes the red–green axis, with positive values indicating a shift toward red and negative values toward green; the b* axis reflects the yellow–blue dimension, where positive values indicate yellow and negative values indicate blue. The C* value represents chroma, (color saturation), indicating the vividness or intensity of the color. The hue angle (h°) represents the actual shade of the color, with 0° corresponding to red, 90° to yellow, 180° to green, and 270° to blue (Kulapichitr, Borompichaichartkul, Fang, Suppavorasatit, & Cadwallader, 2022).

**Fig. 1:**
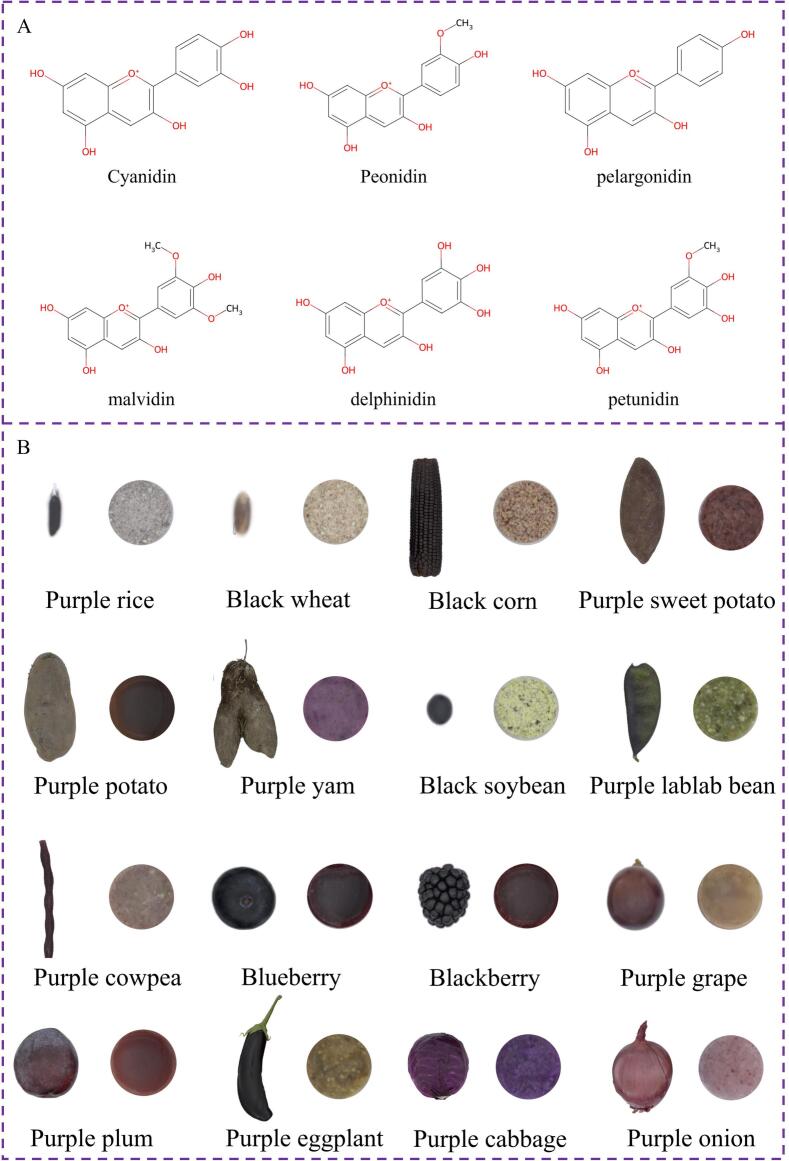
Structures of common anthocyanidins (A) and *E*-eye images of representative purple/black plant foods surface and after homogenization (B). (For interpretation of the references to color in this figure legend, the reader is referred to the web version of this article.)

Although purple/black agricultural products often exhibit similar surface colors, the color characteristics of the homogenized samples differ notably from their external appearance (Fig. 1). Overall, homogenized samples showed reduced chroma and generally darker tones (Table 1). Among them, 10 samples displayed a purplish hue, 4 exhibited a yellowish tone, and 2 appeared greenish in color. The L* values ranged from 18.27 to 71.07, indicating significant variation in lightness and brightness among the different samples. Black soybean, black wheat, and purple rice showed markedly higher L* values compared to the others, suggesting relatively lighter colors after homogenization. In contrast, samples such as purple potato, blueberry, and blackberry exhibited lower L* values, indicating darker coloration. The a* values across all samples ranged from −2.17 to 21.48. Except for black soybean and purple lablab bean, which had negative a* values indicating a greenish hue, all other samples exhibited predominantly reddish tones. Purple onion had the highest a* value, reflecting the most intense red coloration among all samples. The b* values ranged from −13.07 to 23.63. Purple cabbage, purple yam, and purple potato showed negative b* values, suggesting a shift toward blue. The remaining samples exhibited positive b* values. Notably, black soybean, eggplant, purple lablab bean, and grape showed significantly higher b* values than the others, indicating a predominantly yellowish tone after homogenization. The C* values of all samples ranged from 1.11 to 25.17, indicating that most samples exhibited low color saturation and generally dark tones, particularly in the case of blackberry and blueberry. Regarding the hue angle (h°), the values ranged from 7.93° to 346.61° and were mostly concentrated in two regions: below 45° and above 270°. Specifically, 10 samples had hue angles between 0° and 90°. Among them, purple rice, purple sweet potato, purple cowpea, blueberry, blackberry, purple plum, and purple onion had h° values below 45°, indicating a coloration leaning toward reddish-purple. The hue angles of black wheat, black corn, purple grape, and purple eggplant fell within the 50°–70° range, suggesting a shift toward yellowish tones. Black soybean and purple lablab bean had hue angles close to 90°, with respectively values of 94.85° and 96.03°, indicating a dominant green hue. In contrast, purple potato, purple yam, and purple cabbage exhibited h° values greater than 270°, suggesting a color tendency toward blue or even violet tones.

**Table 1:** CIE LAB color space values of homogenized samples of purple/black agricultural products.

Samples	L*	a*	b*	C*	h
Purple rice	59.39 ± 0.59a	4.54 ± 0.48h	1.44 ± 0.29i	4.90 ± 0.40i	17.36 ± 1.50de
Black wheat	67.12 ± 0.29b	8.81 ± 0.02g	14.30 ± 0.30e	16.79 ± 0.25g	58.38 ± 0.56cd
Black corn	50.36 ± 0.19d	14.49 ± 0.05d	18.35 ± 0.30d	23.38 ± 0.20bc	51.70 ± 0.54cde
Purple sweet potato	31.14 ± 0.23j	17.12 ± 0.40b	9.33 ± 0.16f	19.49 ± 0.40e	28.59 ± 0.48de
Purple potato	18.27 ± 1.22m	3.43 ± 0.90i	−1.10 ± 1.04k	4.14 ± 1.24i	282.04 ± 135.48b
Purple yam	36.57 ± 0.21i	17.79 ± 0.52b	−4.24 ± 0.24l	18.28 ± 0.52f	346.61 ± 0.75a
Black bean	71.07 ± 1.12a	−2.00 ± 0.27 k	23.63 ± 0.44a	23.72 ± 0.44bc	94.83 ± 0.64c
Purple lablab bean	39.29 ± 0.81h	−2.17 ± 0.30k	21.97 ± 0.86bc	22.09 ± 0.88d	96.03 ± 1.11c
Purple cowpea	45.09 ± 0.24g	10.51 ± 0.48f	9.30 ± 0.24f	14.04 ± 0.52h	41.53 ± 0.55de
Blueberry	20.98 ± 0.89l	1.08 ± 0.46j	0.15 ± 0.19j	1.11 ± 0.43j	7.93 ± 0.00e
Blackberry	22.22 ± 1.1l	3.67 ± 0.75hi	1.47 ± 0.24i	3.96 ± 0.76i	22.09 ± 3.34de
Purple grape	47.19 ± 1.47f	13.41 ± 0.42e	21.30 ± 0.92c	25.17 ± 0.83c	57.78 ± 1.28cd
Purple plum	26.39 ± 1.07k	15.75 ± 2.02c	7.63 ± 1.15g	17.50 ± 2.32g	17.81 ± 2.45de
Purple eggplant	40.67 ± 2.96h	10.53 ± 0.63f	22.66 ± 1.28bc	25.00 ± 1.10bc	65.04 ± 2.05cd
Purple cabbage	26.76 ± 0.34k	4.27 ± 0.93hi	−13.07 ± 0.74m	13.77 ± 0.92m	287.96 ± 3.15b
Purple onion	48.51 ± 0.84e	21.48 ± 0.77a	6.85 ± 0.31h	22.54 ± 0.79h	17.68 ± 0.66de

The values are the mean ± standard deviation, different letter (a–m) in the same row indicate significant differences (p < 0.05).

### 3.2 Chlorophyll analysis in purple/black agricultural products

Chlorophyll is a complex green pigment abundantly found in plants, algae, and certain bacteria, where it plays an essential role in photosynthesis and constitutes an important component of the human diet (Martins, Barros, Rosa, & Antunes, 2023). As shown in Table 2, chlorophyll B was not detected in any of the samples. However, relatively high concentrations of chlorophyll A were detected in black soybean and purple lablab bean, reaching 102.38 μg/g and 781.41 μg/g, respectively. A low level of chlorophyll A was also detected in purple eggplant (3.64 μg/g). Collectively, these findings suggest that the presence of chlorophyll may contribute to the color expression of black soybean, purple lablab bean, and purple eggplant.

**Table 2:** Pigment contents in different purple/black agricultural products.

Samples	Chlorophyll A (μg/g)	Lutein (μg/g)	Carotene (μg/g)	Total anthocyanins (μg/g)
Purple rice	ND	1.29 ± 0.01g	0.22 ± 0.01f	676.58 ± 8.35c
Black wheat	ND	0.22 ± 0.00j	ND	7.80 ± 2.55l
Black corn	ND	4.35 ± 0.02c	0.25 ± 0.01e	53.46 ± 3.34jk
Purple sweet potato	ND	0.20 ± 0.00j	0.12 ± 0.00g	389.80 ± 6.96ef
Purple potato	ND	1.29 ± 0.01g	0.43 ± 0.01c	358.06 ± 10.07fg
Purple yam	ND	ND	0.04 ± 0.00i	75.18 ± 6.02j
Black bean	102.38 ± 0.13b	11.38 ± 0.07a	0.09 ± 0.00h	35.64 ± 5.87kl
Purple lablab bean	781.41 ± 3.93a	7.85 ± 0.04b	1.09 ± 0.03a	45.66 ± 5.10jk
Purple cowpea	ND	1.38 ± 0.01f	0.08 ± 0.00h	170.95 ± 5.37h
Blueberry	ND	3.44 ± 0.02d	0.41 ± 0.01c	1441.13 ± 7.53a
Blackberry	ND	2.08 ± 0.01e	0.59 ± 0.02b	1029.62 ± 55.16b
Purple grape	ND	1.28 ± 0.01g	0.42 ± 0.01c	589.71 ± 8.35d
Purple plum	ND	0.51 ± 0.00i	0.39 ± 0.01d	345.25 ± 10.87g
Purple eggplant	3.64 ± 0.02c	0.97 ± 0.01h	0.27 ± 0.01e	133.64 ± 9.30i
Purple cabbage	ND	ND	ND	403.16 ± 56.04e
Purple onion	ND	0.09 ± 0.00k	ND	75.73 ± 10.21j

The values are the mean ± standard deviation, different letter (a–l) in the same row indicate significant differences (p < 0.05) and “ND” indicates not detected.

### 3.3 Carotenoid analysis in purple/black agricultural products

Carotenoids are a widely distributed group of tetraterpenoid pigments in nature, typically imparting yellow, orange, or red coloration (Maoka, 2020). In this study, the contents of lycopene, lutein, and carotene (including both α-carotene and β-carotene) were measured in the 16 samples. The results showed that lycopene was not detected in any of the samples, while lutein and carotene were present in most of them. As shown in the Table 2, significant differences in lutein and carotene content were observed among the samples. Except for purple yam and purple cabbage, all other samples contained detectable levels of lutein. The highest lutein content was found in black soybean (11.38 μg/g), followed by purple lablab bean (7.85 μg/g) and black corn (4.35 μg/g). Carotene was not detected in black wheat, purple cabbage, and purple onion. Among the samples containing carotene, purple lablab bean exhibited the highest level (1.09 μg/g), which was significantly greater than that observed in the other samples. In summary, the overall carotenoid content in purple/black agricultural products was relatively low. Among the carotenoids analyzed, lutein was the predominant compound.

### 3.4 Anthocyanin analysis in purple/black agricultural products

#### 3.4.1 Total anthocyanin content analysis

Anthocyanins contribute to a wide range of colors including orange, red, purple, blue, and nearly black (Marin & Pucker, 2023), and are widely regarded as the principal contributors to the purple/black appearance of many agricultural products. Table 2 illustrates the total anthocyanin content in the 16 samples. The results revealed notable differences in anthocyanin content despite all samples exhibiting a purple/black surface color. Blueberry and blackberry showed significantly higher anthocyanin levels than the other samples, reaching 1441.13 μg/g and 1029.62 μg/g, respectively. Moderate levels of anthocyanins were observed in purple rice, purple grape, purple cabbage, purple sweet potato, purple potato, purple plum, purple cowpea, and purple eggplant, with concentrations ranging from 133.64 to 676.58 μg/g. In contrast, purple onion, purple yam, black corn, purple lablab bean, black soybean, and black wheat had relatively low anthocyanin contents, all below 100 μg/g. Among them, black wheat exhibited the lowest concentration at only 7.80 μg/g.

#### 3.4.2 Analysis of anthocyanin composition

**Fig. 2:**
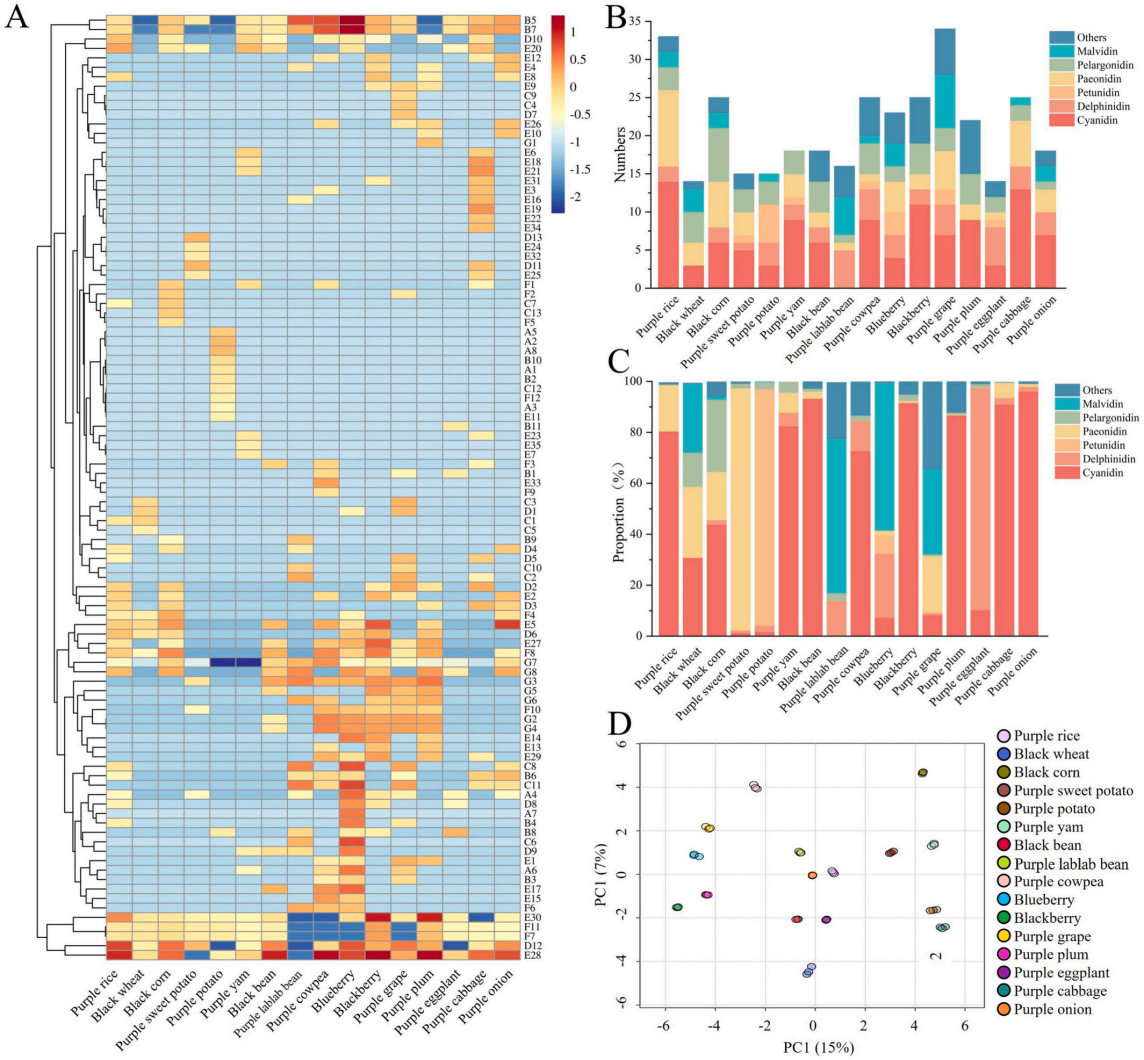
Anthocyanin composition analysis of purple/black agricultural products. (A) Heatmap of anthocyanin composition; (B) Number of identified anthocyanin compounds; (C) Proportional distribution of anthocyanin types; (D) Principal component analysis (PCA) of anthocyanin profiles across purple/black plant foods. (For interpretation of the references to color in this figure legend, the reader is referred to the web version of this article.)

Through interactions with other compounds, anthocyanins form more than 600 different derivatives currently known worldwide. Nevertheless, only six anthocyanidin types (cyanidin, delphinidin, petunidin, peonidin, pelargonidin, and malvidin) are widely distributed in nature, accounting for approximately 90% of identified anthocyanins (Enaru, Drecanu, Pop, & Diaconeasa, 2021). Based on HPLC-MS/MS analysis, a total of 92 anthocyanins and 8 other compounds were identified. Among them, 35 were cyanidin derivatives, followed by 13 peonidin derivatives, 11 delphinidin derivatives, 12 pelargonidin derivatives, 13 malvidin derivatives, and 8 petunidin derivatives. As shown in Fig. 2A and Table S2, the compositional characteristics of these anthocyanins varied significantly among different purple/black agricultural products, with particularly notable variation observed in purple sweet potato. Certain characteristic compounds exhibited a pronounced enrichment trend in specific samples, indicating species-specific diversity in anthocyanin metabolic pathways and accumulation mechanisms.

Regarding the number of anthocyanin and related compounds detected in each sample (Fig. 2B), purple grape exhibited the greatest diversity, containing all six types of anthocyanins as well as six additional compounds, totaling 34 identified substances. In addition, purple cowpea and blueberry also covered all six anthocyanidin types, with 25 and 23 compounds identified, respectively (including other substances). Purple rice (33 total compounds), purple corn (25), purple cabbage (25), and purple onion (18) contained five types of anthocyanins, lacking only petunidin derivatives. In contrast, purple yam (18), purple sweet potato (15), and purple eggplant (14) lacked malvidin derivatives, while purple potato (15) lacked peonidin derivatives. Among the samples containing four types of anthocyanins, blackberry (25) and black soybean (18) both lacked petunidin and malvidin derivatives. Purple lablab bean (16) was missing petunidin and cyanidin derivatives, while black wheat (14) lacked petunidin and delphinidin derivatives. Purple plum (22) was the only sample that contained just three types of anthocyanins—cyanidin, peonidin, and pelargonidin derivatives.

Based on the proportional distribution of anthocyanins and related compounds in each sample (Fig. 2C), cyanidin derivatives were detected in 15 of the 16 samples, with the exception of purple lablab bean. They represented the most structurally diverse group among all anthocyanins and were the dominant components in most samples, accounting for 72.75% to 96.12% of total anthocyanins in purple rice, purple yam, black soybean, purple cowpea, blackberry, purple plum, purple cabbage, and purple onion. Peonidin derivatives were present in all samples except purple potato and predominated in purple sweet potato, accounting for 95.07% of total anthocyanins. Delphinidin derivatives occurred in 14 samples, absent only in black wheat and purple plum, which made up 87.18% of the total anthocyanins in purple eggplant. In contrast, petunidin and malvidin derivatives were less prevalent, being detected in only 10 and 7 samples, respectively. Notably, petunidin derivatives were predominant in purple potato, accounting for 92.74% of the total. Malvidin derivatives were abundant in purple lablab bean and blueberry, comprising 60.47% and 57.92%, respectively. Proanthocyanidins accounted for a relatively high proportion in grape (34.31%), whereas isorhamnetin and naringenin were detected at low levels in all samples and were not regarded as major constituents. Overall, cyanidin derivatives were the most representative class of anthocyanins. Delphinidin and peonidin derivatives were also widely distributed and dominant in specific samples. Although pelargonidin derivatives were present in all samples, their content was generally low. Petunidin and malvidin derivatives played a major role only in a few specific samples. These findings are consistent with previous studies (Enaru et al., 2021; He & Giusti, 2010).

To further characterise differences in anthocyanin composition among various purple/black agricultural products, principal component analysis (PCA) was performed to evaluate within-group consistency and between-group discrimination. This method provides a comprehensive integrated view of the overall metabolic differences and sample variation. As shown in Fig. 2D, the three biological replicates within each group were highly overlapped, indicating good reproducibility and analytical stability of the UPLC–MS/MS-based anthocyanin detection method. Meanwhile, clear separation was observed among different sample groups, suggesting significant differences in both anthocyanin composition and content across the purple/black agricultural products. Principal components PC1 and PC2 explained 15% and 7% of the total variance, respectively. Notably, fruit samples (purple grape, blueberry, purple plum, and blackberry) and tuber samples (purple potato, purple sweet potato, and purple yam) formed relatively clustered distributions in the PCA score plot, with considerable separation between the two categories. This indicates a certain degree of compositional similarity within each category, but significant differences between fruits and tubers. Although legume (black bean, purple lablab bean, and purple cowpea) and vegetable (purple eggplant, purple cabbage, and purple onion) samples were generally distributed in a similar region of the PCA space, noticeable heterogeneity was still observed. By contrast, cereal samples (black corn, purple rice, and black wheat) showed a more dispersed distribution, reflecting a higher level of heterogeneity in their anthocyanin composition.

#### 3.4.3 Analysis of dominant anthocyanin composition

**Fig. 3:**
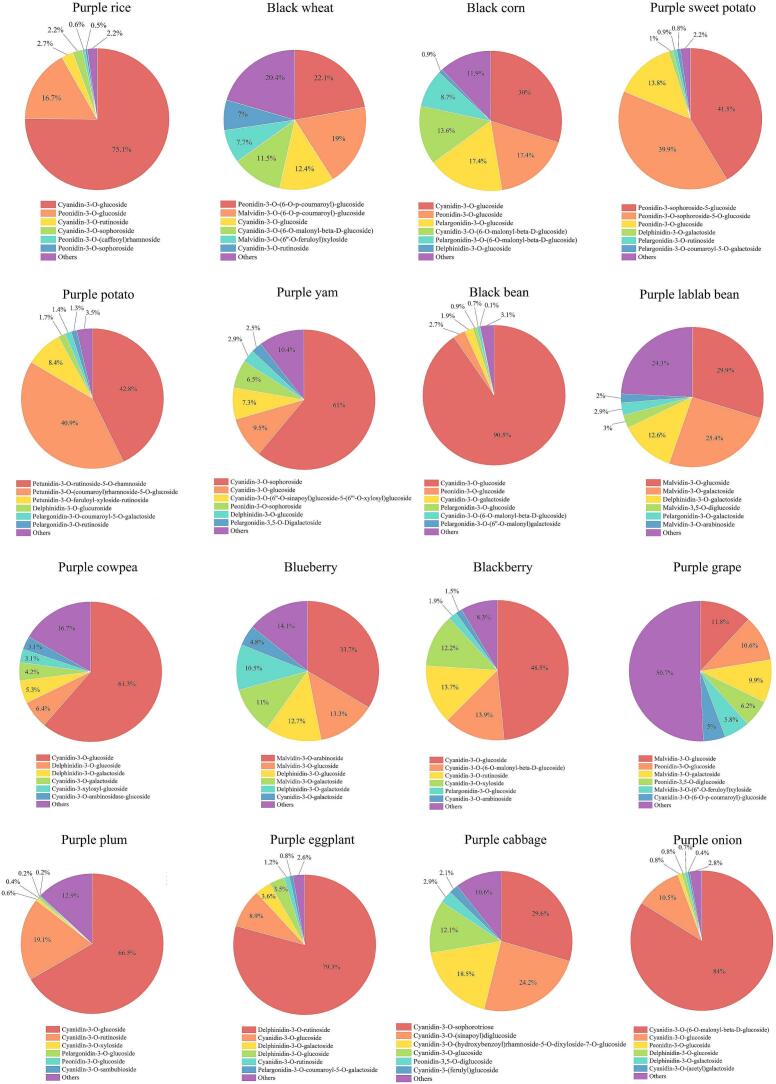
Relative contents of the six predominant anthocyanins in each purple/black agricultural product. (For interpretation of the references to color in this figure legend, the reader is referred to the web version of this article.)

To further investigate the compositional differences in the major anthocyanins among purple/black agricultural products, the six most abundant anthocyanins in each sample were selected for proportional analysis. As shown in Fig. 3 and Table S2, the dominant anthocyanin structures differed significantly among sample types and displayed clear species-specific characteristics.

Among cereal samples, purple rice was dominated by cyanidin-type anthocyanins, with Cyanidin-3-O-glucoside and Peonidin-3-O-glucoside together accounting for as much as 91.8%, indicating a highly concentrated anthocyanin profile. In black wheat, a high proportion of acylated anthocyanins was observed, primarily Peonidin-3-O-(6-O-p-coumaroyl)-glucoside and Malvidin-3-O-(6-O-p-coumaroyl)-glucoside, each contributing approximately 20%. In black corn, 3-O-glucoside structures predominated, with Cyanidin-3-O-glucoside, Peonidin-3-O-glucoside, and Pelargonidin-3-O-glucoside as the major constituents, which together accounted for 64.8% of the total anthocyanin content.

Among tuber samples, purple sweet potato was dominated by peonidin-type anthocyanins. Peonidin-3-sophoroside-5-glucoside, Peonidin-3-O-sophoroside-5-O-glucoside, and Peonidin-3-O-glucoside together accounted for 95.2% of the total anthocyanin content. In purple potato, petunidin-type anthocyanins were predominant, comprising more than 92.1% of the total. The major compounds included Petunidin-3-O-rutinoside-5-O-rhamnoside, Petunidin-3-O-(coumaroyl) rhamnoside-5-O-glucoside, and Petunidin-3-O-feruloyl-xyloside-rutinoside. In purple yam, cyanidin-type anthocyanins were the dominant group, accounting for at least 77.8%, with Cyanidin-3-O-sophoroside alone contributing 61.0%.

Among the legume samples, black bean was primarily rich in Cyanidin-3-O-glucoside, which accounted for as much as 90.0% of the total anthocyanin content, followed by Peonidin-3-O-glucoside at only 2.7%. In purple lablab bean, malvidin-type anthocyanins were dominant, with Malvidin-3-O-glucoside and Malvidin-3-O-galactoside together comprising over 50% of the total. In purple cowpea, Cyanidin-3-O-glucoside was also the major component, accounting for more than 60%, while the remaining anthocyanins were distributed more evenly.

Among the fruit samples, blueberry was dominated by malvidin- and delphinidin-type anthocyanins, with Malvidin-3-O-arabinoside being the most abundant, accounting for 33.7% of the total. Blackberry primarily contained cyanidin-type anthocyanins, including Cyanidin-3-O-glucoside (48.5%), Cyanidin-3-O-(6-O-malonyl-beta-D-glucoside) (13.9%), Cyanidin-3-O-rutinoside (13.7%), and Cyanidin-3-O-xyloside (2.2%). Purple grape exhibited a rich diversity of anthocyanins, however, no single compound dominated. Instead, the composition was relatively balanced, with proanthocyanidins accounting for a substantial proportion. In purple plum, Cyanidin-3-O-glucoside was the major anthocyanin, representing 66.5% of the total, followed by Cyanidin-3-O-rutinoside at 19.1%.

Among the vegetable samples, delphinidin-type anthocyanins were predominant in purple eggplant, accounting for more than 86.4% of the total, with Delphinidin-3-O-rutinoside alone contributing 79.3%. In purple cabbage, cyanidin-type anthocyanins were dominant, with the four most abundant cyanidin derivatives collectively accounting for 84.4% of the total anthocyanin content. Purple onion was also dominated by cyanidin-type anthocyanins, with Cyanidin-3-O-(6-O-malonyl-beta-d-glucoside) being the most abundant, representing 84.0% of the total.

Overall, Cyanidin-3-O-glucoside was the predominant anthocyanin in most samples, highlighting its widespread occurrence and relative stability in purple agricultural products. In terms of structural types, anthocyanins were mainly present in glucoside-conjugated forms, which provide the molecular basis for their water solubility and stability. These findings are generally consistent with previous studies (He & Giusti, 2010).

### 3.5 Antioxidant activity analysis of purple/black agricultural products

**Fig. 4:**
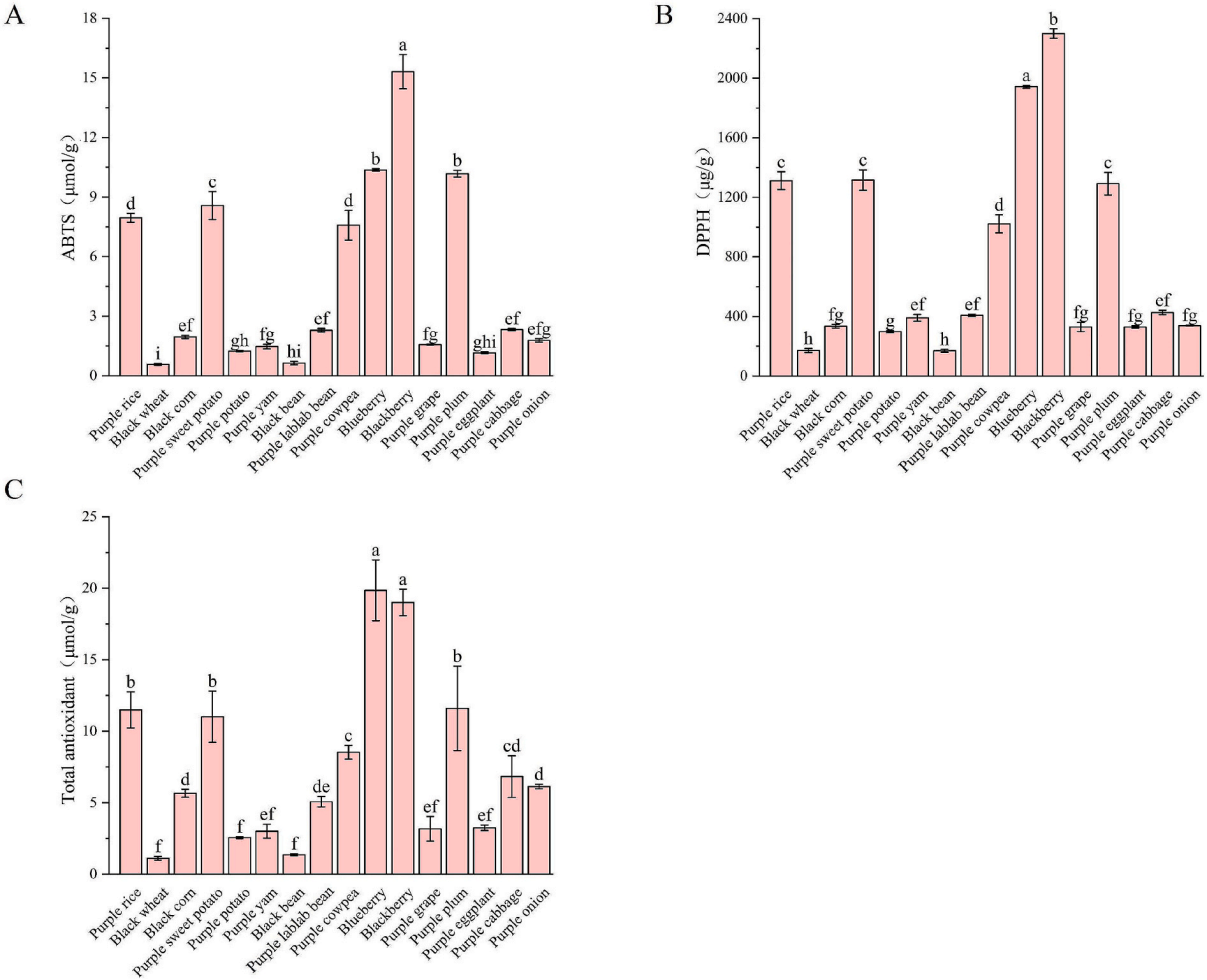
Antioxidant activity of different purple/black agricultural products. (A) ABTS radical scavenging capacity; (B) DPPH radical scavenging capacity; (C) Total antioxidant capacity. (For interpretation of the references to color in this figure legend, the reader is referred to the web version of this article.)

ABTS and DPPH radical scavenging capacities, along with TAC, are commonly used indicators for evaluating in vitro antioxidant activity of samples and have been widely applied in functional food research (Zhang et al., 2025). As shown in Fig. 4A, in terms of ABTS radical scavenging activity, blackberry exhibited the strongest performance (15.32 μmol/g), which was significantly higher than that of the other samples (*p* < 0.05). Blueberry and purple plum ranked next, with values of 10.36 μmol/g and 10.18 μmol/g. Additionally, purple rice, purple sweet potato, and purple cowpea also demonstrated relatively high ABTS scavenging capacities, reaching 7.95 μmol/g, 8.58 μmol/g, and 7.58 μmol/g, respectively. Regarding DPPH radical scavenging activity, blackberry and blueberry again showed significant advantages, with values of 2299.92 μg/g and 1943.11 μg/g (Fig. 4B). These were followed by purple sweet potato (1316.01 μg/g), purple rice (1311.32 μg/g), and purple plum (1291.41 μg/g). As shown in Fig. 4C, blueberry and blackberry also exhibited significantly higher total antioxidant capacities than the other samples (*p* < 0.05), reaching 19.86 μmol/g and 19.01 μmol/g. They were followed by purple plum (11.61 μmol/g), purple rice (11.49 μmol/g), and purple sweet potato (11.02 μmol/g). In summary, marked differences in antioxidant capacity were observed among the various purple agricultural products. Blackberry and blueberry consistently outperformed superior performance across all three antioxidant indicators, highlighting their strong potential as natural antioxidant sources. Purple sweet potato, purple rice, plum, and purple cowpea also showed relatively high antioxidant activity.

### 3.6 Correlation analysis among color parameters, pigments, and antioxidant activity

**Fig. 5:**
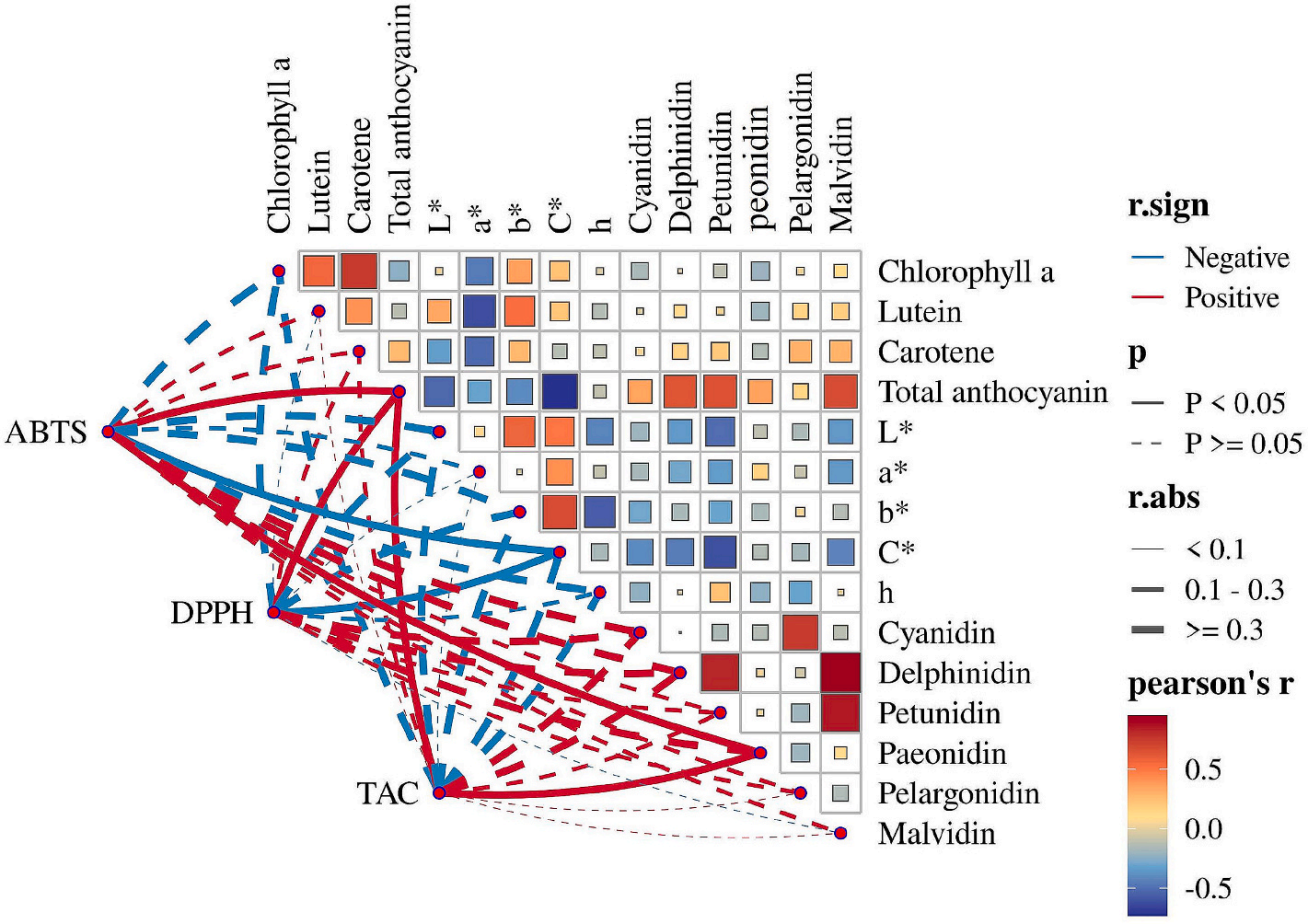
Correlation analysis among color parameters, pigment composition, and antioxidant activity in purple/black agricultural products. (For interpretation of the references to color in this figure legend, the reader is referred to the web version of this article.)

Correlation analysis was conducted to evaluate the relationships among color parameters, pigment composition, and antioxidant capacity in purple/black agricultural products. As shown in Fig. 5, the L* value was significantly positively correlated with the C* value (*p* < 0.05), indicating that darker colors generally exhibited lower chroma. Both L* and C* values were significantly negatively correlated with total anthocyanin content (*p* < 0.01), suggesting that higher anthocyanin levels are associated with deeper, darker coloration. Among individual anthocyanin types, petunidin-type anthocyanins showed a significant negative correlation with L* value (p < 0.05). Delphinidin-type anthocyanins also exhibited a negative correlation with L* value. In addition, chlorophyll A content was significantly positively correlated with b* value (p < 0.01) and negatively correlated with a* value. Lutein content was significantly negatively correlated with a* value (p < 0.01), suggesting its association with yellow-green color tones. In terms of antioxidant activity, ABTS and DPPH radical scavenging capacities, as well as TAC, were all highly significantly positively correlated with total anthocyanin content (p < 0.01). Among the anthocyanin types, ABTS and TAC showed significant correlations with cyanidin- and peonidin-type anthocyanins, indicating that these two classes are the primary contributors to antioxidant activity. Further analysis revealed that the L* and C* values were significantly negatively correlated with all three antioxidant indicators (p < 0.01), suggesting that samples with darker color and lower chroma tend to exhibit stronger antioxidant capacity. This conclusion is consistent with previous findings on the functional properties of anthocyanins (He & Giusti, 2010).

## 4 Discussion

The color of food is typically determined by its natural pigments, which not only provide distinct visual characteristics but also offer various health benefits, including antioxidant, anti-inflammatory, anticancer, and immunomodulatory effects (Gui et al., 2023). As such, food color can serve as an indicator of specific nutritional components and bioactive compounds present in the product (Kumar et al., 2020). In recent years, purple/black agricultural products have garnered increasing attention due to growing consumer awareness of nutrition (Francavilla & Joye, 2020; Nuszkiewicz et al., 2025). However, theoretical understanding of the relationship between color and nutritional quality in these products remains limited. Investigating the associations between color and nutritional attributes of agricultural products can not only help consumers make informed dietary choices based on visual cues and personal nutritional needs, but also provide scientific evidence for governments to refine dietary guidelines and develop nutrition policies. In this study, we examined the color characteristics, pigment composition, and antioxidant capacity of 16 purple/black agricultural products, analyzed their interrelationships, and preliminarily explored the potential link between color and nutritional quality in such products.

The study found that although the surface colors of purple/black agricultural products appeared similar, differences in tissue structure and pigment distribution resulted in clear discrepancies between surface appearance and the color characteristics of homogenized samples. The homogenized samples exhibited a range of colors including purple, yellow, and green. Nevertheless, anthocyanins remained the primary contributors to the purple/black appearance in most products, although chlorophyll A and carotenoids played a modifying role in certain samples such as black soybean and purple lablab bean. Our results of the present study show a certain degree of consistency with previous reports (Chumanova, Efremova, Sobolev, & Kosyaeva, 2025; Im, Kim, & Lee, 2021; Paulsmeyer et al., 2017; Quan et al., 2019; Sahamishirazi, Moehring, Claupein, & Graeff-Hoenninger, 2017; Sharma et al., 2018; Shibata, Ohara, Matsumoto, Hasegawa, & Akimoto, 2021; Wiczkowski, Szawara-Nowak, & Topolska, 2015; Zheng et al., 2022). Significant differences in anthocyanin content and composition were observed among the different samples. Previous studies have also demonstrated that different cultivars and geographical origins of the same agricultural product can markedly influence anthocyanin content and composition, which is closely associated with varietal characteristics, pigment distribution patterns, regulatory mechanisms of pigment biosynthesis, and environmental factors (Enaru et al., 2021). In general, the genetic background of a species or cultivar determines the types of anthocyanins present, while environmental factors influence the accumulation levels of individual anthocyanins in different ways (Jaakola, 2013). For example, blueberry and blackberry exhibited the highest total anthocyanin contents, which may be attributed to the upregulation of structural genes involved in anthocyanin biosynthesis during fruit ripening (Jaakola et al., 2002; Wu et al., 2024). In contrast, black wheat had the lowest total anthocyanin content, potentially due to the action of transcription factors such as MYBL2, which suppress anthocyanin accumulation by downregulating biosynthetic genes (Shoeva, Gordeeva, & Khlestkina, 2014). The diversity and abundance of anthocyanins are closely associated with the genetic background of the species, the regulatory capacity of transcription factors (e.g., MYB, bHLH, and WD40), and the expression levels of structural genes in the biosynthetic pathway, including CHS, DFR, ANS, and UFGT (Chen, Pinson, Jackson, & Edwards, 2023). In terms of anthocyanin diversity, purple grape showed the greatest number of anthocyanin types, likely due to the polymorphism within the VvMYBA gene family (Jaakola, 2013). However, some samples exhibited a relatively narrow anthocyanin profile. For instance, purple potato was dominated by petunidin derivatives, which may be explained by high expression of the F3′5′H enzyme (Jinggui, Sudisha, Le, Xin, & Mostafa, 2018). In contrast, purple sweet potato contained 95.07% peonidin-type anthocyanins, indicating more active expression of F3′H (Jinggui et al., 2018). In addition, cyanidin-type anthocyanins were the predominant components in most samples, while delphinidin-, malvidin-, and petunidin-type anthocyanins appeared in a species-specific manner. This is primarily due to the widespread expression of F3′H, the enzyme responsible for cyanidin biosynthesis, in many plant species (Jeong, Goto-Yamamoto, Hashizume, & Esaka, 2006). Owing to the complexity of anthocyanin biosynthetic regulation, distinct differences in anthocyanin composition were observed among samples. Even within the same category (e.g., fruits and cereals), purple/black agricultural products exhibited both compositional similarities or variability. Beyond gene expression, external environmental factors such as climate conditions, soil type, and light intensity in the growing region may also influence the biosynthesis and accumulation of anthocyanins to some extent (Jeong et al., 2006). In summary, the compositional differences and diversity of anthocyanins among purple/black agricultural products are fundamentally driven by expression patterns of key genes in the anthocyanin biosynthetic pathway, the diversity of structural modifications (e.g., glycosylation, acylation, and methylation), and the specificity and complexity of regulatory networks. In addition, pH, enzyme activities, and environmental growing conditions jointly modulate anthocyanin biosynthesis and accumulation. (Sunil & Shetty, 2022).

Correlation analysis indicated that both L* and C* values were significantly negatively correlated with total anthocyanin content. Notably, L* showed particularly strong inverse associations with petunidin- and delphinidin-type anthocyanins. In terms of anthocyanin coloration, higher concentrations typically result in darker appearance. Moreover, increased B-ring hydroxylation tends to shift hues toward blue, whereas methylation typically induces a red shift. (Glover & Martin, 2012). For instance, although purple sweet potato and purple potato exhibited similar total anthocyanin levels, the latter displayed darker and more bluish hue. This is attributed to the dominance of petunidin-type anthocyanins in purple potato, in contrast to the peonidin-type anthocyanins predominant in purple sweet potato. Since petunidin contains more hydroxyl groups on the B-ring than peonidin, its pigmentation is deeper and more violet–blue (Alappat & Alappat, 2020). The ABTS, DPPH radical scavenging capacities, and TAC of the samples were all highly significantly positively correlated with total anthocyanin content. Among the different types, cyanidin- and peonidin-type anthocyanins showed strong correlations with ABTS and TAC values. Previous studies have shown that the antioxidant activity of anthocyanins depends not only on their concentration but also on structural determinants, particularly the degree of B-ring hydroxylation and overall molecular simplicity. Monosaccharide-conjugated anthocyanins exhibit stronger antioxidant capacity than polysaccharide-conjugated forms, and glycosylated anthocyanins are generally more active than acylated forms (Dudek et al., 2022). Purple grape was characterized by malvidin-type anthocyanins and contained higher total anthocyanin levels than purple plum, its antioxidant activity was comparatively lower. In contrast, purple plum—dominated by cyanidin-type anthocyanins—showed comparatively stronger antioxidant performance. Similarly, purple sweet potato was dominated by peonidin-type anthocyanins and exhibited stronger antioxidant activity than purple potato and purple cabbage, even though they showed comparable total anthocyanin levels. This may be attributed to the simpler anthocyanin structures in purple sweet potato, which contain fewer sugar moieties and acylation modifications (Gao et al., 2022). Overall, both L* and C* values were significantly negatively correlated with ABTS and DPPH radical scavenging capacities as well as TAC, indicating that samples with darker colors tend to have stronger antioxidant potential. This is likely because anthocyanins, the major pigments responsible for the coloration of purple/black agricultural products, range in hue from red to purplish-blue and possess strong antioxidant properties (Zhu et al., 2024). Anthocyanin content contributes significantly to both the visual darkness and the antioxidant activity of the samples. However, in addition to content, anthocyanin type, structure, distribution, as well as factors such as pH and the presence of other compounds (e.g., metal ions, phenolic acids), can also greatly influence both color intensity and antioxidant capacity (Enaru et al., 2021). However, in addition to content, anthocyanin type, structure, distribution, as well as factors such as pH and the presence of other compounds (e.g., metal ions, phenolic acids), can also greatly influence both color intensity and antioxidant capacity (Enaru et al., 2021). Nevertheless, the present study mainly focused on bulk compositional analysis and correlation-based interpretation, without systematically examining the tissue-specific distribution of anthocyanins or their interactions with matrix-related factors.

Therefore, future research could focus on investigating the distribution patterns of anthocyanins within plant tissues, with particular attention to elucidating the synergistic mechanisms by which anthocyanin structural types influence both coloration and antioxidant capacity in purple/black agricultural products. This includes an integrated analysis of external factors such as pH, metal ions, and co-existing phenolic acids, which may regulate both color stability and functional activity. Establishing a comprehensive correlation model that incorporates these variables would further enhance our understanding of the complex interactions underlying anthocyanin functionality.

## 5 Conclusion

This study provided a comprehensive analysis of the color characteristics, pigment composition, and antioxidant-related correlations in 16 representative purple/black agricultural products. The results revealed that although the surface colors of these products appeared similar, differences in internal structure and pigment distribution led to noticeable discrepancies between their surface and homogenized color characteristics. The homogenized samples generally exhibited darker shades, with most displaying a purple hue, a few leaning toward yellow, and two appearing green. Pigment content analysis showed that except for black soybean and purple lablab bean, which had relatively high levels of chlorophyll A, anthocyanins were the primary pigments responsible for coloration in the remaining samples, with blueberry and blackberry exhibiting the highest total anthocyanin contents. In terms of anthocyanin composition, a diverse range of anthocyanins was identified, with purple grape containing the most types. Cyanidin-type anthocyanins were the most widely distributed and served as the dominant type in most samples—particularly cyanidin-3-O-glucoside. Heatmap and clustering analyses indicated that the anthocyanin composition varied across different categories (e.g., cereals, tubers, legumes, fruits, and vegetables), and certain characteristic compounds were highly enriched in specific samples. Among the different product categories, three cereals exhibited the greatest heterogeneity in anthocyanin composition, followed by three legumes and three vegetables. The anthocyanin profiles of four fruits and three tubers showed greater compositional similarity. In terms of antioxidant capacity, significant differences were observed among the samples, with blueberry and blackberry demonstrating the strongest antioxidant activities. Correlation analysis revealed that total anthocyanin content was significantly negatively correlated with L* and C* values, and highly significantly positively correlated with antioxidant capacity (ABTS and TAC), indicating that higher anthocyanin levels were associated with darker sample coloration and stronger antioxidant activity. Petunidin- and delphinidin-type anthocyanins showed negative correlations with lightness (L*), while cyanidin- and peonidin-type anthocyanins were positively correlated with antioxidant indicators. These findings suggest that in addition to anthocyanin content, anthocyanin type and structural characteristics also influence both coloration and antioxidant potential in purple/black agricultural products.

## CRediT authorship contribution statement

**Xiao Ren:** Writing – review & editing, Writing – original draft, Methodology, Formal analysis, Conceptualization. **Jianfeng Pu:** Investigation, Formal analysis. **Ying Nie:** Software, Methodology. **Yuchen Wang:** Writing – review & editing, Investigation. **Shuxian Huang:** Visualization, Methodology. **Dazhou Zhu:** Writing – review & editing, Project administration.

## Declaration of competing interest

The authors declare that they have no known competing financial interests or personal relationships that could have appeared to influence the work reported in this paper.

## Data Availability

Data will be made available on request.
