# Supplementary material for: Association analysis of color characteristics, anthocyanin profiles, and antioxidant capacity in purple/black agricultural products based on targeted metabolomics
Source: Food Chem X. 2026 Aug 2;38:104268. doi: 10.1016/j.fochx.2026.104268 (PMC13475680; doi:10.1016/j.fochx.2026.104268)
Supplement: Supplementary file 1 [file mmc1.docx]

**Supplementary Material**

Table S1. Information on the selected purple agricultural products.

| **Sample Name** | **Variety** | **Source** | **Place of Origin** |
| --- | --- | --- | --- |
| Purple rice | Self-produced variety | Yao Duoduo, Shanghai Xinji Food Co., Ltd. | Xishuangbanna, Yunnan Province, China |
| Black wheat | Black Pearl No. 1 | Local variety | Xinxiang, Henan Province, China |
| Black corn | Self-produced variety | Dole (Shanghai) Fruit and Vegetable Trading Co., Ltd. | Gongzhuling, Jilin Province, China |
| Purple sweet potato | Jinguiba(Self-produced variety) | Zhongshan Kangsha Food Manufacturing Co., Ltd. | Chengmai, Hainan Province, China |
| Purple potato | Heijingang | Local variety | Tengzhou, Shandong Province, China |
| Purple yam | Ziyu | Local variety | Ganzhou, Jiangxi Province, China |
| Black soybean | Fuhei No. 2 | Local variety | Shenyang, Liaoning Province, China |
| Purple lablab bean | Dongtai local purple lablab bean | Local variety | Yancheng, Jiangsu Province, China |
| Purple cowpea | Suzijiang No. 2 | Local variety | Jiangsu Province, China |
| Blueberry | Sekoya Pop | Local variety | Peru |
| Blackberry | Self-produced variety | Driscoll’s Inc. | Kunming, Yunnan Province, China |
| Purple Grape | Kyoho | Local variety | Jinhua, Zhejiang Province, China |
| Purple Plum | Dinosaur Egg | Local variety | United States |
| Purple Eggplant | Ziyang | Local variety | Shandong Province, China |
| Purple cabbage | Primero | Local variety | Lanzhou, Gansu Province, China |
| Purple Onion | Minle Ziguan | Local variety | Lanzhou, Gansu Province, China |

Table S2. The information of anthocyanin identified in UPLC–MS/MS.

| **No.** | **Compounds** | **Molecular Weight** | **Ionization model** | **CAS** | **Content (μg/g, Fresh weight)** | | | | | | | | | | | | | | | |
| --- | --- | --- | --- | --- | --- | --- | --- | --- | --- | --- | --- | --- | --- | --- | --- | --- | --- | --- | --- | --- |
|  |  |  |  |  | **Purple rice** | **Black wheat** | **Black corn** | **Purple sweet potato** | **Purple potato** | **Purple yam** | **Black bean** | **Purple lablab bean** | **Purple cowpea** | **Blueberry** | **Blackberry** | **Purple grape** | **Purple plum** | **Purple eggplant** | **Purple cabbage** | **Purple onion** |
| **Peonidin** | |  |  |  |  |  |  |  |  |  |  |  |  |  |  |  |  |  |  |  |
| A1 | Petunidin-3-O-(feruloyl)rutinoside-5-O-glucoside | 963.28 | [M]+ | - | - | - | - | - | 0.07±0.00 | - | - | - | - | - | - | - | - | - | - | - |
| A2 | Petunidin-3-O-(coumaroyl)rhamnoside-5-O-glucoside | 771.21 | [M]+ | - | - | - | - | - | 4.90±0.29 | - | - | - | - | - | - | - | - | - | - | - |
| A3 | Petunidin-3-O-(galloyl)(acetyl)(xylosyl)xyloside-5-O-glucoside | 937.22 | [M]+ | - | - | - | - | - | 0.01±0.00 | - | - | - | - | - | - | - | - | - | - | - |
| A4 | Petunidin-3-O-arabinoside | 449.11 | [M]+ | 749848-37-1 | 0.01±0.00 | - | - | 0.01±0.00 | - | - | - | 0.03±0.00 | - | 252.79±8.89 | 0.1±0.03 | 0.05±0.01 | - | 0.02±0.00 | - | 0.01±0.00 |
| A5 | Petunidin-3-O-feruloyl-xyloside-rutinoside | 933.27 | [M]+ | - | - | - | - | - | 1.02±0.16 | - | - | - | - | - | - | - | - | - | - | - |
| A6 | Petunidin-3-O-galactoside | 479.12 | [M]+ | - | - | - | - | - | - | 0.01±0.00 | - | - | 0.36±0.01 | 303.61±4.35 | - | 0.96±0.03 | - | - | - | - |
| A7 | Petunidin-3-O-glucoside | 479.12 | [M]+ | 6988-81-4 | - | - | - | - | - | - | - | - | - | 758.14±56.74 | - | - | - | - | - | - |
| A8 | Petunidin-3-O-rutinoside-5-O-rhamnoside | 771.23 | [M]+ | - | - | - | - | - | 5.15±0.66 | - | - | - | - | - | - | - | - | - | - | - |
| **Total** |  |  |  |  | 0.01±0.00 | - | - | 0.01±0.00 | 11.14±1.11 | 0.01±0.00 | - | 0.03±0.00 | 0.36±0.01 | 1314.53±59.2 | 0.10±0.03 | 1.01±0.03 | - | 0.02±0.00 | - | 0.01±0.00 |
| **Delphinidin** | |  |  |  |  |  |  |  |  |  |  |  |  |  |  |  |  |  |  |  |
| B1 | Delphinidin-3,5-O-diglucoside | 627.16 | [M]+ | 17670-06-3 | - | - | - | - | - | - | - | - | 0.35±0.01 | - | - | 0.01±0.00 | - | 0.03±0.00 | - | - |
| B2 | Delphinidin-3-(6''-O-coumaroyl)rutinoside-5-O-glucoside | 919.25 | [M]+ | - | - | - | - | - | 0.06±0.00 | - | - | - | - | - | - | - | - | - | - | - |
| B3 | Delphinidin-3-O-(6-O-acetyl)-glucoside | 507.11 | [M]+ | 66594-00-1 | - | - | - | - | - | - | - | - | 0.02±0.00 | 0.69±0.07 | - | 0.27±0.02 | - | - | - | - |
| B4 | Delphinidin-3-O-arabinoside | 435.09 | [M]+ | 324533-67-7 | 0.02±0 | - | - | - | - | - | - | - | - | 342.88±18.28 | - | 0.04±0.01 | - | - | - | - |
| B5 | Delphinidin-3-O-galactoside | 465.10 | [M]+ | 197250-28-5 | 0.38±0.03 | - | 1.27±0.12 | 0.18±0.03 | - | 0.20±0.04 | 0.16±0.02 | 71.77±1.12 | 99.06±2.10 | 1929.22±117.46 | 4.04±0.53 | 0.71±0.07 | - | 0.37±0.01 | 2.05±0.25 | 7.79±0.55 |
| B6 | Delphinidin-3-O-sophoroside | 627.16 | [M]+ | 59212-40-7 | 0.02±0 | - | - | - | - | - | - | 0.19±0.02 | 0.53±0.03 | 0.44±0.01 | - | 0.01±0.00 | - | - | 1.01±0.04 | 4.76±0.16 |
| B7 | Delphinidin-3-O-glucoside | 465.10 | [M]+ | 50986-17-9 | 0.42±0.07 | - | 1.46±0.03 | - | - | 0.25±0.04 | 0.15±0.04 | 5.08±0.11 | 119.29±1.97 | 2337.77±168.48 | 4.60±0.58 | 0.76±0.06 | - | 0.35±0.03 | 6.73±0.26 | 9.12±0.52 |
| B8 | Delphinidin-3-O-rutinoside | 611.16 | [M]+ | 15674-58-5 | - | - | - | - | 0.04±0.01 | - | - | 0.63±0.17 | - | 0.06±0.00 | 0.03±0.00 | - | - | 8.03±0.602 | - | - |
| B9 | Delphinidin-3-O-rutinoside-5-O-glucoside | 759.20 | [M]+ | - | - | - | - | - | - | - | - | 2.30±0.67 | - | - | - | - | - | - | - | - |
| B10 | Delphinidin-3-O-glucuronide | 479.08 | [M]+ | - | - | - | - | - | 0.20±0.01 | - | - | - | - | - | - | - | - | - | - | - |
| B11 | Delphinidin-caffeoyl-rutinoside | 773.19 | [M]+ | - | - | - | - | - | - | - | - | - | - | - | - | - | - | 0.04±0 | - | - |
| **Total** |  |  |  |  | 0.84±0.10 | - | 2.73±0.15 | 0.18±0.03 | 0.3±0.02 | 0.46±0.02 | 0.31±0.03 | 79.97±1.57 | 219.26±3.61 | 4611.06±303.17 | 8.67±1.11 | 1.81±0.11 | - | 8.82±0.63 | 9.79±0.42 | 21.66±1.00 |
| **Malvidin** |  |  |  |  |  |  |  |  |  |  |  |  |  |  |  |  |  |  |  |  |
| C1 | Malvidin-3-O-(6-O-p-coumaroyl)-glucoside | 639.17 | [M]+ | 34693-53-3 | 0.07±0.01 | 0.68±0.06 | - | - | - | - | - | - | - | - | - | - | - | - | - | - |
| C2 | Malvidin-3,5-O-diglucoside | 655.19 | [M]+ | - | - | - | - | - | - | - | - | 17.52±2.64 | - | - | - | 10.41±0.76 | - | - | 0.01±0.00 | - |
| C3 | Malvidin-3-O-(6''-O-feruloyl)xyloside | 639.17 | [M]+ | - | - | 0.27±0.00 | - | - | - | - | - | - | - | - | - | 12.51±0.42 | - | - | - | - |
| C4 | Malvidin-3-O-(6''-acetylglucoside)-5-glucoside | 697.20 | [M]+ | - | - | - | - | - | - | - | - | - | - | - | - | 1.42±0.03 | - | - | - | - |
| C5 | Malvidin-3-O-5-O-(6-O-coumaroyl)-diglucoside | 801.22 | [M]+ | - | - | 0.01±0.00 | - | - | - | - | - | - | - | - | - | - | - | - | - | - |
| C6 | Malvidin-3-O-arabinoside | 463.12 | [M]+ | 785047-89-4 | - | - | - | - | - | - | - | 11.73±0.66 | - | 6200.44±715.94 | - | - | - | - | - | - |
| C7 | Malvidin-3-O-(6''-O-feruloyl)glucoside | 669.18 | [M]+ | - | 0.01±0 | - | 0.94±0.05 | - | - | - | - | - | - | - | - | - | - | - | - | - |
| C8 | Malvidin-3-O-galactoside | 493.13 | [M]+ | 30113-37-2 | 0.14±0.03 | - | - | - | - | - | - | 146.14±17.60 | - | 2015.79±175.68 | - | 21.26±0.80 | - | - | - | 0.18±0.02 |
| C9 | Malvidin-3-O-(caffeoyl)glucoside-5-O-glucoside | 817.22 | [M]+ | - | - | - | - | - | - | - | - | - | - | - | - | 0.34±0.02 | - | - | - | - |
| C10 | Malvidin-3-O-xyloside | 463.12 | [M]+ | - | - | - | - | - | - | - | - | 0.77±0.03 | - | - | - | 0.42±0.01 | - | - | - | - |
| C11 | Malvidin-3-O-glucoside | 493.13 | [M]+ | 18470-06-9 | - | - | - | - | - | - | - | 171.79±18.64 | 0.32±0.04 | 2446.26±242.05 | - | 25.37±1.03 | - | - | 0.18±0.03 | 0.21±0.01 |
| C12 | Malvidin-3-O-rhamnoside-5-O-diglucoside-7-O-glucoside | 963.30 | [M]+ | - | - | - | - | - | 0.02±0.00 | - | - | - | - | - | - | - | - | - | - | - |
| C13 | Malvidin-3-O-(6''-O-sinapoyl)xyloside | 669.18 | [M]+ | - | - | - | 1.03±0.02 | - | - | - | - | - | - | - | - | - | - | - | - | - |
| **Total** |  |  |  |  | 0.22±0.03 | 0.96±0.06 | 1.97±0.07 | - | 0.02±0.00 | - | - | 347.94±39.20 | 0.32±0.04 | 10662.49±1128.45 | - | 71.74±1.62 | - | - | 0.19±0.03 | 0.38±0.03 |
| **Peonidin** |  |  |  |  |  |  |  |  |  |  |  |  |  |  |  |  |  |  |  |  |
| D1 | Peonidin-3-O-(6-O-p-coumaroyl)-glucoside | 609.16 | [M]+ | - | - | 0.79±0.14 | - | - | - | - | - | - | - | 0.01±0 | - | 4.72±0.43 | - | - | - | - |
| D2 | Peonidin-3,5-O-diglucoside | 625.18 | [M]+ | 47851-83-2 | 0.68±0.10 | - | 1.20±0.02 | - | - | - | - | - | - | - | 0.04±0.01 | 13.38±0.43 | 0.13±0.01 | - | 11.24±0.42 | - |
| D3 | Peonidin-3,5-diglucoside | 625.18 | [M]+ | - | 0.38±0.05 | - | 0.66±0.04 | - | - | - | - | - | - | - | - | - | 0.07±0 | - | 5.99±0.42 | 0.9±0.06 |
| D4 | Peonidin-3-O-(6''-O-malonyl)diglucoside | 711.18 | [M]+ | - | 0.03±0.01 | - | 0.11±0.01 | - | - | - | - | 0.03±0.00 | - | - | - | - | - | - | - | 1.92±0.07 |
| D5 | Peonidin-3-O-(6-O-p-coumaroyl)-galactoside | 609.16 | [M]+ | - | 0.01±0.00 | - | - | - | - | - | - | - | - | - | - | 3.92±0.21 | - | - | 1.19±0.11 | - |
| D6 | Peonidin-3-O-(caffeoyl)rhamnoside | 609.16 | [M]+ | - | 3.46±0.60 | 0.09±0.01 | 0.33±0.03 | - | - | - | - | - | - | 2.4±0.21 | 13.24±0.27 | - | 2.4±0.07 | - | - | - |
| D7 | Peonidin-3-O-5-O-(6-O-coumaroyl)-diglucoside | 771.21 | [M]+ | - | - | - | - | - | - | - | - | - | - | - | - | 2.44±0.09 | - | - | - | - |
| D8 | Peonidin-3-O-arabinoside | 433.11 | [M]+ | - | 0.01±0.00 | - | - | - | - | - | - | - | - | 72.7±9.42 | 0.06±0.01 | - | - | - | - | - |
| D9 | Peonidin-3-O-galactoside | 463.12 | [M]+ | - | - | - | - | - | - | 0.02±0.01 | 0.14±0.04 | 0.24±0.04 | - | 189.38±19.33 | - | - | - | - | - | - |
| D10 | Peonidin-3-O-sophoroside | 625.18 | [M]+ | - | 2.72±0.47 | - | 0.08±0.01 | - | - | 0.56±0.02 | 0.02±0.00 | - | 0.05±0.01 | 0.09±0.00 | 0.01±0.00 | - | 0.01±0.00 | - | 0.22±0.01 | - |
| D11 | Peonidin-3-O-sophoroside-5-O-glucoside | 787.23 | [M]+ | - | - | - | - | 7.12±0.46 | - | - | - | - | - | - | - | - | - | - | 4.53±0.11 | - |
| D12 | Peonidin-3-O-glucoside | 463.12 | [M]+ | 68795-37-9 | 91.98±17.38 | 0.12±0.03 | 27.25±0.78 | 2.45±0.48 | - | 0.09±0.00 | 15.06±0.58 | - | 0.67±0.02 | 65.63±4.23 | 4.32±0.68 | 22.71±0.63 | 4.28±0.22 | - | 0.26±0.03 | 9.83±0.21 |
| D13 | Peonidin-3-sophoroside-5-glucoside | 787.23 | [M]+ | 79549-69-2 | - | - | - | 7.41±0.32 | - | - | - | - | - | - | - | - | - | - | - | - |
| **Total** |  |  |  |  | 99.28±18.62 | 1.00±0.14 | 29.63±0.87 | 16.99±0.31 | - | 0.67±0.02 | 15.22±0.61 | 0.27±0.04 | 0.72±0.02 | 330.21±33.14 | 17.67±0.93 | 47.17±1.39 | 6.89±0.3 | - | 23.43±0.97 | 12.65±0.18 |
| **Cyanidin** |  |  |  |  |  |  |  |  |  |  |  |  |  |  |  |  |  |  |  |  |
| E1 | Cyanidin-3-O-(6-O-p-coumaroyl)-glucoside | 595.15 | [M]+ | - | - | - | - | - | - | - | - | - | 0.04±0.01 | 0.11±0.02 | - | 10.78±0.4 | 2.65±0.11 | - | - | - |
| E2 | Cyanidin-3,5-O-diglucoside | 611.16 | [M]+ | 20905-74-2 | 0.81±0.14 | - | 0.14±0.02 | - | - | - | - | - | 0.25±0.04 | - | 6.17±0.29 | 0.09±0.00 | - | - | - | 3.61±0.03 |
| E3 | Cyanidin-3-O-(6''-O-ferulyl-xylosyl)glucoside | 757.20 | [M]+ | - | - | - | - | - | - | - | - | - | 0.01±0.00 | - | - | - | - | - | 3.21±0.39 | - |
| E4 | Cyanidin-3-O-(6''-O-malonyl)galactoside | 535.11 | [M]+ | - | - | - | - | - | - | - | - | 0.05±0.00 | - | - | 0.32±0.06 | - | 0.03±0.00 | - | - | 3.96±0.42 |
| E5 | Cyanidin-3-O-(6-O-malonyl-beta-D-glucoside) | 535.11 | [M]+ | 171828-62-9 | 0.98±0.15 | 0.42±0.12 | 21.21±0.86 | - | - | - | 3.67±0.19 | - | 9.43±0.8 | 0.27±0.08 | 378.84±13.7 | - | 0.43±0.13 | - | - | 975.57±16.71 |
| E6 | Cyanidin-3-O-(6''-O-sinapoyl)glucoside-5-(6'''-O-xylosyl)glucoside | 949.26 | [M]+ | - | - | - | - | - | - | 0.63±0.14 | - | - | - | - | - | - | - | - | 1.28±0.09 | - |
| E7 | Cyanidin-3-O-(6''-O-acetyl)-rutinoside | 637.18 | [M]+ | - | - | - | - | - | - | 0.04±0 | - | - | - | - | - | - | - | - | - | - |
| E8 | Cyanidin-3-O-(6''-O-acetyl-2''-O-xylosyl)glucoside | 623.16 | [M]+ | - | 0.26±0.05 | - | - | - | - | - | - | - | - | - | 3.19±0.09 | - | 0.02±0.00 | - | - | 0.11±0.01 |
| E9 | Cyanidin-3-O-(6''-O-caffeoyl)rhamnoside | 595.15 | [M]+ | - | - | - | - | - | - | - | - | - | - | - | 0.08±0.01 | 0.88±0.09 | 0.11±0.00 | - | - | - |
| E10 | Cyanidin-3-O-(malonyl)(glucoside)galactoside | 697.16 | [M]+ | - | - | - | - | - | - | - | - | - | - | - | - | - | 0.06±0.01 | - | - | 2.66±0.07 |
| E11 | Cyanidin-3-(coumaryl-ferulyl)glucoside | 771.19 | [M]+ | - | - | - | - | - | 0.01±0.00 | - | - | - | - | - | - | - | - | - | - | - |
| E12 | Cyanidin-3-O-(acetyl)galactoside | 491.12 | [M]+ | - | - | - | - | - | - | - | - | - | 0.09±0.01 | - | 1.2±0.01 | - | - | - | 0.04±0.01 | 5.17±0.55 |
| E13 | Cyanidin-3-[6''-(rhamnosyl)glucoside] | 595.17 | [M]+ | - | - | - | - | - | - | - | - | - | 0.1±0.01 | - | 3.74±0.08 | - | 1.72±0.20 | - | - | - |
| E14 | Cyanidin-3-O-arabinoside | 419.10 | [M]+ | - | - | - | - | - | - | - | - | - | - | 321.6±30.83 | 40.79±1.75 | - | 0.82±0.17 | - | - | - |
| E15 | Cyanidin-3-O-arabinosidase-glucoside | 581.15 | [M]+ | - | - | - | - | - | - | - | - | - | 57.56±3.07 | 0.93±0.06 | - | - | - | - | - | - |
| E16 | Cyanidin-3-(ferulyl)glucoside | 625.16 | [M]+ | - | - | - | - | - | - | - | - | 0.01±0.00 | - | - | - | - | - | - | 8.35±0.97 | - |
| E17 | Cyanidin-3-O-galactoside | 449.11 | [M]+ | 142506-26-1 | - | - | - | - | - | - | 10.74±1.07 | - | 77.82±3.35 | 872.67±56.26 | - | - | - | - | - | - |
| E18 | Cyanidin-3-O-(hydroxybenzoyl)rhamnoside-5-O-dixyloside-7-O-glucoside | 979.27 | [M]+ | - | - | - | - | - | - | 0.1±0.01 | - | - | - | - | - | - | - | - | 72.58±4.12 | - |
| E19 | Cyanidin-3-O-sophorotriose | 773.21 | [M]+ | - | - | - | - | - | - | - | - | - | - | - | - | - | - | - | 115.95±3.12 | - |
| E20 | Cyanidin-3-O-sophoroside | 611.16 | [M]+ | 38820-68-7 | 11.92±2.24 | - | 0.09±0 | 0.02±0 | - | 5.27±0.48 | 0.27±0.05 | - | - | 0.14±0.02 | - | - | - | 0.01±0.00 | 2.43±0.16 | - |
| E21 | Cyanidin-3-O-(sinapoyl)diglucoside | 817.22 | [M]+ | - | - | - | - | - | - | 0.12±0.01 | - | - | - | - | - | - | - | - | 94.81±5.32 | - |
| E22 | Cyanidin-3-O-(sinapoyl)gentianoside | 817.22 | [M]+ | - | - | - | - | - | - | - | - | - | - | - | - | - | - | - | 3.15±0.12 | - |
| E23 | Cyanidin-3-O-(sinapoyl)xyloside-5-O-glucoside-7-O-glucoside | 949.26 | [M]+ | - | - | - | - | - | - | 0.05±0.00 | - | - | - | - | - | - | - | - | 0.02±0.01 | - |
| E24 | Cyanidin-3-sinapoyl-xyloside-glucoside-galactoside | 949.26 | [M]+ | - | - | - | - | 0.03±0.00 | - | - | - | - | - | - | - | - | - | - | - | - |
| E25 | Cyanidin-3-O-caffeoyl-glucoside-5-O-sophoroside | 935.25 | [M]+ | - | - | - | - | 0.04±0.00 | - | - | - | - | - | - | - | - | - | - | 0.43±0.02 | - |
| E26 | Cyanidin-3-gentiobioside | 611.16 | [M]+ | - | - | - | - | - | - | - | - | - | 0.22±0.03 | - | - | 0.08±0.01 | 0.52±0.03 | - | - | 3.66±0.11 |
| E27 | Cyanidin-3-O-xyloside | 419.10 | [M]+ | 29761-24-8 | 0.18±0.03 | - | 0.03±0.01 | - | - | - | 0.41±0.03 | - | 2.1±0.09 | 8.09±0.80 | 334.03±4.68 | 0.14±0.04 | 11.76±0.50 | - | - | - |
| E28 | Cyanidin-3-O-glucoside | 449.11 | [M]+ | 47705-70-4 | 413.26±75.36 | 0.44±0.05 | 46.84±1.55 | - | 0.1±0.03 | 0.81±0.05 | 499.37±30.08 | - | 1145.14±27.79 | 149.78±15.7 | 1324.01±50.75 | 5.63±1.6 | 1218.55±54.24 | 0.9±0.16 | 47.45±6.62 | 121.65±2.66 |
| E29 | Cyanidin-3-O-sambubioside | 581.15 | [M]+ | 63535-17-1 | - | - | - | - | - | - | - | - | 8.68±0.83 | 0.2±0.01 | 31.93±1.15 | - | 3.4±0.16 | - | 0.03±0.01 | - |
| E30 | Cyanidin-3-O-rutinoside | 595.17 | [M]+ | 28338-59-2 | 14.66±2.72 | 0.25±0.05 | 0.25±0.03 | 0.11±0.02 | 0.10±0.01 | 0.07±0.02 | 0.34±0.02 | - | - | 0.54±0.02 | 373.63±12.06 | 0.25±0.06 | 349.37±13.05 | 0.12±0.01 | - | 0.09±0.01 |
| E31 | Cyanidin-3-O-rutinoside-glucoside | 757.22 | [M]+ | - | - | - | - | - | - | - | - | - | - | - | 0.02±0.00 | - | - | - | 3.19±0.28 | - |
| E32 | Cyanidin-3-diglucoside-5-glucuronide | 787.19 | [M]+ | - | - | - | - | 0.02±0.00 | - | - | - | - | - | - | - | - | - | - | - | - |
| E33 | Cyanidin-3-xylosyl-glucoside | 581.15 | [M]+ | - | - | - | - | - | - | - | - | - | 58.02±1.42 | - | - | - | - | - | - | - |
| E34 | Cyanidin-sinapoyl-galloyl-malonyl-glucoside-sambubioside | 1187.27 | [M]+ | - | - | - | - | - | - | - | - | - | - | - | - | - | - | - | 3.88±0.26 | - |
| E35 | Cyanidin-rutinoside-rhamnoside | 741.22 | [M]+ | - | - | - | - | - | - | 0.02±0.00 | - | - | - | - | - | - | - | - | - | - |
| **Total** |  |  |  |  | 442.07±80.68 | 1.11±0.22 | 68.55±1.64 | 0.23±0.02 | 0.21±0.02 | 7.12±0.58 | 514.81±30.87 | 0.07±0.00 | 1359.46±22.18 | 1354.33±103 | 2497.96±61.95 | 17.85±2.2 | 1589.44±68.15 | 1.04±0.17 | 356.81±5.12 | 1116.48±13.91 |
| **Pelargonidin** | |  |  |  |  |  |  |  |  |  |  |  |  |  |  |  |  |  |  |  |
| F1 | Pelargonidin-3,5-O-Digalactoside | 595.17 | [M]+ | - | - | - | 1.30±0.01 | - | - | 0.21±0.02 | - | - | 0.1±0.02 | - | - | - | - | - | 0.01±0.00 | - |
| F2 | Pelargonidin-3,5-O-diglucoside | 595.17 | [M]+ | - | - | - | 1.00±0.06 | - | - | - | - | - | - | - | - | 0.05±0.00 | - | - | - | - |
| F3 | Pelargonidin-3-O-(6''-O-malonyl)galactoside | 519.11 | [M]+ | - | - | - | - | - | - | - | 0.74±0.07 | - | 0.58±0.03 | - | - | - | - | - | 0.01±0.00 | - |
| F4 | Pelargonidin-3-O-(6-O-malonyl-beta-D-glucoside) | 519.11 | [M]+ | 165070-68-8 | 0.04±0.01 | 0.03±0 | 13.64±0.62 | - | - | - | - | - | - | 0.01±0.00 | - | - | - | - | - | 0.16±0.01 |
| F5 | Pelargonidin-3-O-(dimalonyl)glucoside | 565.16 | [M]+ | - | - | - | 0.15±0.01 | - | - | - | - | - | - | - | - | - | - | - | - | - |
| F6 | Pelargonidin-3-O-galactoside | 433.11 | [M]+ | 197451-24-4 | - | - | - | - | - | - | - | 16.48±3.11 | 3.61±0.54 | 3.93±0.55 | - | - | - | - | - | - |
| F7 | Pelargonidin-3-O-coumaroyl-5-O-galactoside | 579.15 | [M]+ | - | 0.29±0.03 | 0.22±0.04 | 0.43±0.02 | 0.15±0.02 | 0.17±0.03 | 0.07±0.02 | 0.11±0.01 | - | - | - | 8.25±1.52 | - | 2.05±0.14 | 0.08±0.01 | 0.06±0.01 | 0.05±0.01 |
| F8 | Pelargonidin-3-O-glucoside | 433.11 | [M]+ | 47684-27-5 | 1.05±0.19 | 0.02±0 | 27.18±0.89 | - | - | - | 5.11±0.60 | - | 29.02±1.63 | 0.72±0.08 | 52.6±7.15 | 0.11±0.00 | 7.38±0.22 | - | - | 0.05±0.01 |
| F9 | Pelargonidin-3-O-sambubioside | 565.16 | [M]+ | - | - | - | - | - | - | - | - | - | 0.09±0.01 | - | - | - | - | - | - | - |
| F10 | Pelargonidin-3-O-rhamnoside-5-O-glucoside | 579.17 | [M]+ | - | - | - | - | 0.01±0.00 | - | - | - | - | 6.55±0.84 | 1.94±0.32 | 0.79±0.16 | 1.13±0.21 | 0.85±0.06 | - | - | - |
| F11 | Pelargonidin-3-O-rutinoside | 579.17 | [M]+ | - | 0.22±0 | 0.21±0.03 | 0.38±0.04 | 0.16±0.02 | 0.15±0 | 0.06±0.02 | 0.1±0.01 | - | - | - | 7.8±1.44 | - | 1.86±0.06 | 0.05±0.01 | 0.06±0.01 | 0.05±0.01 |
| F12 | Pelargonidin-3-O-rutinoside-5-O-glucoside | 741.22 | [M]+ | 202338-79-2 | - | - | - | - | 0.02±0 | - | - | - | - | - | - | - | - | - | - | - |
| **Total** |  |  |  |  | 1.61±0.18 | 0.48±0.07 | 44.07±1.56 | 0.31±0.01 | 0.34±0.03 | 0.35±0.06 | 6.06±0.63 | 16.48±3.11 | 39.95±1.70 | 6.60±0.92 | 69.44±10.05 | 1.3±0.21 | 12.13±0.39 | 0.13±0.02 | 0.15±0.02 | 0.31±0.03 |
| **Others** |  |  |  |  |  |  |  |  |  |  |  |  |  |  |  |  |  |  |  |  |
| G1 | Procyanidin A1 | 576.13 | [M+H]+ | 103883-03-0 | - | - | - | - | - | - | - | - | - | - | - | - | 4.24±1.29 | - | - | - |
| G2 | Procyanidin B1 | 578.14 | [M+H]+ | 20315-25-7 | - | - | - | - | - | - | 0.03±0.01 | - | 106±13.82 | 35.38±5.79 | 20.78±1.13 | 22.71±2.33 | 15.98±2.09 | - | - | - |
| G3 | Procyanidin B2 | 578.14 | [M+H]+ | 29106-49-8 | - | - | - | 0.11±0.02 | - | - | 12.33±2.59 | 78.08±11.83 | 6.85±0.55 | 8.5±1.63 | 32.01±3.67 | 20.32±0.92 | 139.12±9.91 | - | - | - |
| G4 | Procyanidin B3 | 578.14 | [M+H]+ | 23567-23-9 | - | - | - | - | - | - | 0.04±0.01 | - | 122.85±15.7 | 37.64±4.12 | 21.91±1.03 | 24.47±3.73 | 16.31±2.13 | - | - | - |
| G5 | Procyanidin B4 | 578.14 | [M+H]+ | 29106-51-2 | - | - | - | - | - | - | 0.36±0.05 | - | - | - | 56.42±9.58 | 3.24±0.34 | 14.9±2.00 | - | - | - |
| G6 | Procyanidin C1 | 866.21 | [M+H]+ | 37064-30-5 | - | - | - | - | - | - | - | 13.02±3.82 | 1.52±0.28 | - | 1.25±0.19 | 3.15±0.90 | 11.56±3.11 | - | - | - |
| G7 | Naringenin | 272.07 | [M+H]+ | 480-41-1 | 0.08±0.01 | 0.01±0.00 | 0.95±0.12 | 0.02±0.00 | - | - | 0.75±0.19 | 2.84±0.1 | 7.94±0.65 | 0.07±0.01 | 0.16±0.01 | 0.09±0.00 | 0.04±0.00 | 0.03±0.01 | 0.01±0.00 | 0.5±0.02 |
| G8 | Quercetin-3-O-glucoside | 464.10 | [M+H]+ | 21637-25-2 | 3.55±1.03 | - | 8.04±1.91 | - | - | - | 1.52±0.32 | 33.89±2.05 | - | 17.85±2.26 | 2.62±0.48 | - | 18.87±5.55 | 0.07±0.02 | - | 8.16±1.44 |
| **Total** |  |  |  |  | 3.62±1.04 | 0.01±0.00 | 8.99±1.88 | 0.13±0.02 | - | - | 15.03±3.13 | 127.83±15.73 | 245.16±28.33 | 99.44±9.36 | 135.15±10.91 | 73.98±5.85 | 221.03±3.44 | 0.10±0.02 | 0.01±0.00 | 8.67±1.44 |

The values are the mean ± standard deviation

-: not detected
